# Computationally Efficient 2D DOA Estimation with Uniform Rectangular Array in Low-Grazing Angle

**DOI:** 10.3390/s17030470

**Published:** 2017-02-26

**Authors:** Junpeng Shi, Guoping Hu, Xiaofei Zhang, Fenggang Sun, Yu Xiao

**Affiliations:** 1 Air and Missile Defense College, Air Force Engineering University, Xi’an 710051, China; 15667081720@163.com (J.S.); hgp6068@163.com (G.H.); 2 Electronic information college, Nanjing University of Aeronautics and Astronautics, Nanjing 210016, China; zhangxiaofei@nuaa.edu.cn; 3 College of Information Science and Engineering, Shandong Agricultural University, Tai’an 271018, China; sunfg@sdau.edu.cn

**Keywords:** two-dimensional direction of arrival estimation, spatial differencing matrix set, low-grazing angle, information loss

## Abstract

In this paper, we propose a computationally efficient spatial differencing matrix set (SDMS) method for two-dimensional direction of arrival (2D DOA) estimation with uniform rectangular arrays (URAs) in a low-grazing angle (LGA) condition. By rearranging the auto-correlation and cross-correlation matrices in turn among different subarrays, the SDMS method can estimate the two parameters independently with one-dimensional (1D) subspace-based estimation techniques, where we only perform difference for auto-correlation matrices and the cross-correlation matrices are kept completely. Then, the pair-matching of two parameters is achieved by extracting the diagonal elements of URA. Thus, the proposed method can decrease the computational complexity, suppress the effect of additive noise and also have little information loss. Simulation results show that, in LGA, compared to other methods, the proposed methods can achieve performance improvement in the white or colored noise conditions.

## 1. Introduction

Two-dimensional (2D) direction of arrival (DOA) estimation including azimuth and elevation angles with different array geometry has been widely applied in wireless communications, radar and sonar signal processing [1]. Recently, the 2D DOA estimation with uniform rectangular arrays (URAs) has attracted widespread concern [2,3,4,5]. Various algorithms have been developed for improving the estimation performance, such as the multiple signal classification (MUSIC) [6], estimation of signal parameters via rotational invariance techniques (ESPRIT) [7] and the matrix pencil (MP) method [8]. However, in a low-grazing angle (LGA) condition [9,10,11,12,13], the coherency between direct and reflected signals of each target can cause the sample covariance matrix to be rank-deficient. To address this problem, both spatial smoothing [14,15,16,17] and spatial differencing [18,19,20] techniques are developed for 2D DOA estimation with URAs.

For spatial smoothing techniques, Yeh et al. [15] developed the spatial smoothing 2D MUSIC algorithm by using the covariance matrices of overlapping rectangular subarrays and Chen et al. [16] presented an analysis of a special smoothing scheme extended in conjunction with the eigenstructure technique. To reduce the computational complexity, a partial spectral search based method (PSS) is proposed for limiting the searching region into a small sector [21]. Then, a tree structure one-dimensional (1D) algorithm [22] was developed based on a repeated use of the 1D MUSIC algorithm. However, it requires a large number of eigenvalue decompositions (EVDs) and does not perform well under a low signal-to-noise ratio (SNR). For spatial differencing techniques, Liu et al. [18] constructed a new spatial differencing matrix to suppress the white noise by using the difference between the first subarray and the spatial backward subarray. By exploiting the difference between the neighboring forward subarrays and backward subarrays, the method in [19] can suppress the colored noise. Additionally, an asymmetric spatial difference smoothing method [20] was used to reduce noise for coherent sources location, especially when the number of targets is odd.

However, due to the 2D peaks search, 2D or 1D EVD operations, all the aforementioned methods suffer from great computational complexity, especially for a large size of subarrays. In other words, these methods can provide better performance at the cost of great computation. Therefore, we concentrate on reducing the computational complexity caused by the 2D operations, while maintaining a high estimation performance. In this paper, we propose a spatial differencing matrix set (SDMS) method for 2D DOA estimation with URA in LGA. Employing the overlapping column or row subarrays along the *x*- or *y*-direction, we build the SDMSs by rearranging the auto-correlation and cross-correlation matrices in turn among different subarrays. In addition, to suppress the data loss, we only perform difference for the auto-correlations and the cross-correlations are kept completely. Then, the two parameters are estimated independently by using the 1D subspace-based estimation technique, the pair-matching of which is achieved by extracting the diagonal elements of the URA. Simulation results verify the effectiveness of the proposed method.

The advantages of the proposed method can be given as follows:

- The methods in [3,4,15,16,17,21] involve the 2D EVD or 2D peak search, while the proposed method can estimate the parameters with 1D subspace-based estimation techniques.
- The method in [22] can only use the auto-correlations of different subarrays, while the proposed method can use more information including auto-correlations and cross-correlations.
- The spatial differencing techniques in [18,19] perform a difference operation on the whole subarrays, while the proposed method is only for the auto-correlations and the cross-correlations are kept completely. Thus, the SDMS method has little data loss.

The rest of this paper is given as follows. In Section 2, the basic received signal model of the URA in LGA is developed. Then, we derive the SDMS method using a 1D subspace-based estimation technique and achieve pair-matching by extracting the diagonal elements of the URA in Section 3. Simulation results are given in Section 4, and we conclude the whole paper in Section 5.

In this paper, operators ${\left(\cdot \right)}^{T}$, ${\left(\cdot \right)}^{*}$ and ${\left(\cdot \right)}^{H}$ represent transpose, conjugation, and conjugate transpose, respectively. $I_{N}$ denotes an N×N identity matrix and $J_{M}$ denotes an M×M exchange matrix with ones on its anti-diagonal and zeros elsewhere. ⊕ and ⊗ represent Hadamard product and Kronecker product, respectively; diag(·) and blkdiag(·) denote the diagonal matrix or the block diagonal matrix operator. E[·] and vec(·) denote expectation and vectorization, respectively.

## 2. System Model

**Figure 1:**
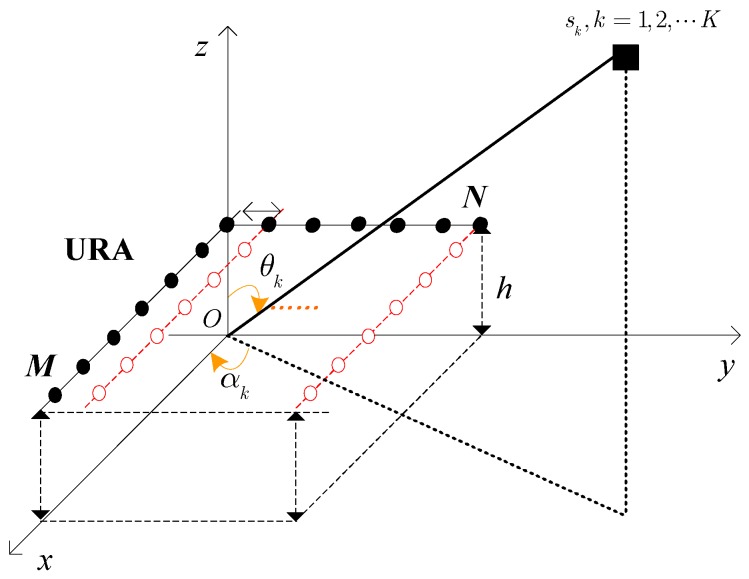
The array geometry model for the URA.

As described in Figure 1, we regard the multipath effect as ideal specular reflection, where both curved earth and atmosphere refraction are not considered. We also consider *K* narrowband far-field signals $s_{k}\left(t\right)$ (k=1,2,⋯,K) impinging on a URA with *MN* well calibrated and identically polarized sensors parallel to the *xoy* plane. Here, both *x*- and *y*- directions of the URA are separated by half a wavelength, the height of which is set as *h*. Since the received signals include two paths, i.e., direct path, reflected path, the output can be given as [11,12]

(1) $$\begin{array}{c} X\left(t\right)=\sum_{k=1}^{K}a_{x}\left(\alpha_{k},\theta_{dk}\right)a_{y}^{T}\left(\alpha_{k},\theta_{dk}\right)s_{k}\left(t\right)+\sum_{k=1}^{K}a_{x}\left(\alpha_{k},\theta_{rk}\right)a_{y}^{T}\left(\alpha_{k},\theta_{rk}\right)\beta_{k}s_{k}\left(t\right)+Z\left(t\right), \end{array}$$

where $\theta_{dk}$ and $\theta_{rk}$ are the direct and reflected elevation angle for the *k*th target ($\theta_{dk}\approx -\theta_{rk}=\theta_{k}$), respectively, $\alpha_{k}$ is the azimuth angle and $\beta_{k}$ is the multipath reflection coefficient from the *k*th signal to receiver array; Let βk=exp[j(π−2πΔRk2πΔRkλλ)], $\Delta R_{k}\approx 2h$$\sin\theta_{k}$ for simplicity, $\Delta R_{k}$ is the difference value between direct path and reflected path in LGA; $a_{x}\left(\alpha_{k},\theta_{k}\right)=a_{x}\left(u_{k}\right)$$={\left[1,e^{-j\pi u_{k}},\ldots ,e^{-j\pi \left(M-1\right)u_{k}}\right]}^{T}$, $a_{y}\left(\alpha_{k},\theta_{k}\right)=a_{y}\left(v_{k}\right)=$ $[1,e^{-j\pi v_{k}},\ldots ,e^{-j\pi \left(N-1\right)v_{k}}{]}^{T}$, $u_{k}=\sin\theta_{k}\cos\alpha_{k}$, and $v_{k}=$ $\sin\theta_{k}\sin\alpha_{k}$. The elements of Z(t) are temporally and spatially complex white Gaussian noises with zero-mean and variance $\sigma^{2}$. Then, by vectorizing the matrix X(t), a composite data vector can be constructed as

(2) $$\begin{array}{c} x\left(t\right)=vec\left(X\left(t\right)\right)=\left(A_{x}\circ A_{y}\right)s\left(t\right)+z\left(t\right), \end{array}$$

where $A_{x}={\left[a_{x}\left(u_{1}\right),a_{x}\left(-u_{1}\right),\cdots ,a_{x}\left(u_{K}\right),a_{x}\left(-u_{K}\right)\right]}^{T}$, $A_{y}=$ $[a_{y}\left(v_{1}\right),a_{y}\left(-v_{1}\right),\cdots ,a_{y}\left(v_{K}\right)$,$a_{y}\left(-v_{K}\right){]}^{T}$, s(t)= $[s_{1}\left(t\right),\beta_{1}s_{1}\left(t\right),$$\cdots ,s_{K}\left(t\right),\beta_{K}s_{K}{\left(t\right)]}_{2K\times 1}^{T}$, z(t)= vec(Z(t)).

By forming $\Phi_{y}$$=diag[e^{-j\pi v_{1}},e^{j\pi v_{1}},\cdots ,e^{-j\pi v_{K}},$$e^{j\pi v_{K}}]$, the model in (2) can be rewritten as

(3) $$\begin{array}{c} x\left(t\right)=\left[\begin{array}{c} x_{1}\\ x_{2}\\ \vdots \\ x_{N} \end{array}\right]=\left[\begin{array}{c} A_{x}\\ A_{x}\Phi_{y}\\ \vdots \\ A_{x}\Phi_{y}^{N-1} \end{array}\right]s\left(t\right)+z\left(t\right). \end{array}$$

From (2) and (3), the incident signals can be divided into *K* parts and the signals in each part are correlated. With *L* snapshots (t=1,2,⋯,L), the sample covariance matrix can be calculated as

(4) $$\begin{array}{c} R_{0}=E\left[x\left(t\right)x^{H}\left(t\right)\right]=\frac{1}{L}\sum_{t=1}^{L}x\left(t\right)x^{H}\left(t\right)=AR_{s}A^{H}+\sigma^{2}I_{MN}, \end{array}$$

where $A=A_{x}\circ A_{y}$, $R_{s}=E\left[s\left(t\right)s^{H}\left(t\right)\right]$ denotes the correlation matrix of coherent signals. Thus, due to rank-deficiency of the covariance matrix $R_{0}$, the classic methods for 2D DOA estimation will lose efficiency [6,7,8].

## 3. 2D DOA Estimation in LGA

In this section, we proposed a SDMS method that uses the differencing matrices among different spatial smoothing subarrays for 2D DOA estimation in LGA, where the parameters $u_{k}$ and $v_{k}$ are estimated independently.

### 3.1. 1D Estimation of Parameter $u_{k}$

**Figure 2:**
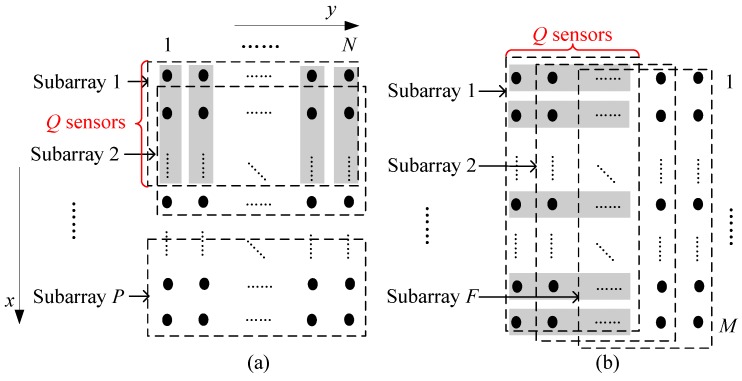
Rectangular subarray grouping of the URA.

Here, as described in Figure 2a, we divide the URA with M×N sensors into *P* overlapping forward rectangular subarrays of size Q×N along the *x*-direction. Each rectangular subarray has *N* column subarrays with *Q* sensors, such as the shaded areas for the first rectangular subarray. Then, the *n*th column of *p*th rectangular subarray can be given as $z_{pn}\left(t\right)=X_{p}y_{n}\left(t\right)=X_{p}G_{n}y\left(t\right)$, where $X_{p}=\left[0_{Q\times (p-1)}\;I_{Q}\;0_{Q\times (P-p)}\right]$, *P* = *M*-*Q* + 1, p=1,2,⋯,P, n=1,2,⋯,N, and $G_{n}=\left[0_{M\times (n-1)M}\;I_{M}\;0_{M\times (N-n)M}\right]$. As a result, using the matrix pencil of auto-correlation and cross-correlation matrices between different column subarrays, we can build the new matrix as

(5) $$\begin{array}{c} R_{xp}=\underset{N\;matrices\;of\;size\;Q\times N}{\underset{︸}{\left\{E\left[z_{p1}z_{p1}^{H}\right],\cdots ,E\left[z_{p1}z_{pN}^{H}\right],\right.}}\underset{N-1\;matrices\;of\;size\;Q\times N}{\underset{︸}{E\left[z_{p2}z_{p2}^{H}\right],\cdots ,E\left[z_{p2}z_{pN}^{H}\right],}}\cdots ,\underset{1}{\underset{︸}{\left.E[z_{pN}z_{pN}^{H}]\right\}}}, \end{array}$$

In (5), because of the calculation of covariance matrices in turn, the data information can be used more effectively. In addition, the cross-correlation matrix $E[z_{pd}z_{pn}^{H}\left(t\right)]$ (d≠n) can also restrain the effect of additive noise. However, the auto-correlation matrix $E[z_{np}z_{np}^{H}\left(t\right)]$ has the noise covariance matrix $\sigma^{2}I_{Q}$, which will certainly decrease the performance. Then, in order to suppress the effect of noise, we first build an initial matrix that has the same noise matrix as $R_{xp}$, and we have

(6) $$\begin{array}{c} C_{x}=\left\{\underset{N\;matrices\;of\;size\;Q\times N}{\underset{︸}{E[z_{11}z_{11}^{H}],0\cdots ,0}},\underset{N-1\;matrices\;of\;size\;Q\times N}{\underset{︸}{E[z_{11}z_{11}^{H}],0,\cdots ,0}},\cdots ,\underset{1}{\underset{︸}{E[z_{11}z_{11}^{H}]}}\right\}. \end{array}$$

Combining (5) and (6), the forward SDMS for the *x*-direction (SDMS-x) can be defined as

(7) $$\begin{array}{c} D_{xp}=C_{x}-J_{Q}R_{xp}^{*}F_{Q}=A_{xQ}{\bar{D}}_{xp}diag\left[A_{xQ}^{H},\cdots ,A_{xQ}^{H}\right], \end{array}$$

where $F_{Q}=blkdiag\left[J_{Q},J_{Q},\cdots ,J_{Q}\right]$, the number of $J_{Q}$ in $F_{Q}$ is N(N+1)/2, ${\bar{D}}_{xp}=\left[R_{s}-\Theta^{Q-p+1}R_{s}^{*}\Theta^{-1+p-Q},\cdots ,-\Theta^{Q-p+1}R_{s}^{*}\Phi^{N-1}\Theta^{-1+p-Q},\cdots ,R_{s}-\Theta^{Q-p+1}\Phi^{1-N}R_{s}^{*}\Phi^{N-1}\Theta^{-1+p-Q}\right]$, $A_{xQ}$ is the submatrix of the array response matrix $A_{x}$ consisting of the first *Q* rows, $X_{p}A_{x}=A_{xQ}\Theta^{p-1}$, and $J_{Q}A_{xQ}^{*}=A_{xQ}\Theta^{Q}$, $\Theta =diag[e^{-j\pi u_{1}},e^{j\pi u_{1}},e^{-j\pi u_{K}},\cdots ,e^{j\pi u_{k}}]$. Following the forward backward (FB) technique, the new FB SDMS-x can be given as

(8) $$\begin{array}{c} D_{x}=\frac{1}{2P}\sum_{p=1}^{P}\left[D_{xp}+J_{Q}{\left(D_{xp}\right)}^{*}F_{Q}\right]. \end{array}$$

Then, based on the definition in (8), we can prove that the new FB SDMS-x has the following property.

**Theorem** **1.** *Assume that there are 2*K *narrowband coherent signals impinging on the URA (M×N sensors). As described in Figure 2a, the URA is divided along the* x*-direction and the number of sensors in each column subarray is* Q*. Then, we define the new FB SDMS-x $D_{x}$ as in (8). If Q≥2K, the rank of $D_{x}$ is equal to the number of the signals, namely, $rank(D_{x})=2$K.*

**Proof.**  See the Appendix A. ☐

Under the Theorem 1, we divide $A_{xQ}$ into two submatrices as $A_{xQ}={\left[A_{xQ1}^{T},A_{xQ2}^{T}\right]}^{T}$, where $A_{xQ1}$ and $A_{xQ2}$ consist of the first 2*K* rows and the last Q−2K rows, respectively. Since the matrix $A_{xQ}$ is a Vandermonde matrix with full rank, we can get a 2K×(Q−2K) linear operator $\Gamma_{x}$ for $A_{xQ2}=\Gamma_{x}^{H}A_{xQ1}$. Then, the matrix $D_{x}$ can be divided into two submatrices as

(9) $$\begin{array}{c} D_{x}=\left[\begin{array}{c} D_{x1}\\ D_{x2} \end{array}\right]\begin{array}{c} \left.\right\}2K\\ \left.\right\}Q-2K \end{array}, \end{array}$$

where $D_{xQ2}=\Gamma_{x}^{H}D_{xQ1}$. In addition, the operator $\Gamma_{x}$ can be calculated as

(10) $$\begin{array}{c} \Gamma_{x}=A_{xQ1}^{-H}A_{xQ2}^{H}={\left(D_{x1}D_{x1}^{H}\right)}^{-1}D_{x1}D_{x2}^{H}. \end{array}$$

Therefore, by constructing the matrix $\Omega_{x}=[\Gamma_{x}^{H},-I_{Q-2K}{]}^{H}$, we can get $\Omega_{x}^{H}A_{xQ}=0_{(Q-2K)\times 2K}$, which can be used to estimate the parameter $u_{k}$. By letting $a\left(u_{k}\right)={\left[1,\;e^{-j\pi u_{k}},\cdots ,\;e^{-j\pi \left(Q-1\right)u_{k}}\right]}^{T}$, the parameter $u_{k}$ can be estimated by minimizing the following cost function

(11) $$\begin{array}{c} f\left(u_{k}\right)=a^{H}\left(u_{k}\right)\Pi_{x}a\left(u_{k}\right), \end{array}$$

where $\Pi_{x}=\Omega_{x}{\left(\Omega_{x}^{H}\Omega_{x}\right)}^{-1}\Omega_{x}^{H}$.

### 3.2. 1D Estimation of Parameter $v_{k}$

Similarly, as described in Figure 2b, we divide the URA into *F* overlapping forward rectangular subarrays along the *y*-direction. Each rectangular subarray has *M* row subarrays with *Q* sensors. Then, the *m*th row of *f*th rectangular subarray can be set as $w_{fm}\left(t\right)=X_{f}G_{m}T_{e}y\left(t\right)$, where $T_{e}$ is the row permutation matrix, $X_{f}=\left[0_{Q\times (f-1)}\;I_{Q}\;0_{Q\times (F-f)}\right]$, f=1,2,⋯,F, m=1,2,⋯,M, *F*=*N*-*Q*+1, and $G_{m}=\left[0_{N\times (m-1)N}\;I_{N}\;0_{N\times (M-m)N}\right]$. Then, we can build the new FB SDMS for the *y*-direction (SDMS-y) as

(12) $$\begin{array}{c} D_{y}=\frac{1}{2F}\sum_{f=1}^{F}\left[D_{yf}+J_{Q}{\left(D_{yf}\right)}^{*}F_{Q}\right], \end{array}$$

where

(13) $$\begin{array}{c} D_{yf}=C_{y}-J_{Q}R_{yf}^{*}F_{Q}=A_{yQ}{\bar{D}}_{yf}diag\left[A_{yQ}^{H},\cdots ,A_{yQ}^{H}\right], \end{array}$$

(14) $$\begin{array}{c} R_{yf}=\underset{M\;matrices\;of\;size\;Q\times M}{\underset{︸}{\left\{E\left[w_{f1}w_{f1}^{H}\right],\cdots ,E\left[w_{f1}z_{fM}^{H}\right],\right.}}\underset{M-1\;matrices\;of\;size\;Q\times M}{\underset{︸}{E\left[w_{f2}w_{f2}^{H}\right],\cdots ,E\left[w_{f2}w_{fM}^{H}\right],}}\cdots ,\underset{1}{\underset{︸}{\left.E[w_{fM}w_{fM}^{H}]\right\}}}, \end{array}$$

(15) $$\begin{array}{c} C_{y}=\left\{\underset{M\;matrices\;of\;size\;Q\times M}{\underset{︸}{E[w_{11}w_{11}^{H}],0\cdots ,0}},\underset{M-1\;matrices\;of\;size\;Q\times M}{\underset{︸}{E[w_{11}w_{11}^{H}],0,\cdots ,0}},\cdots ,\underset{1}{\underset{︸}{E[w_{11}w_{11}^{H}]}}\right\}, \end{array}$$

and $A_{yQ}$ is the submatrix of the array response matrix $A_{y}$ consisting of the first *Q* rows, $X_{f}A_{y}=A_{yQ}\Phi^{f-1}$, $J_{Q}A_{yQ}^{*}=A_{yQ}\Theta^{Q}$, $D_{yf}=[R_{s}-\Phi^{Q-f+1}R_{s}^{*}\Phi^{-1+f-Q},\cdots -\Phi^{Q-f+1}R_{s}^{*}\Theta^{M-1}$$\Phi^{-1+f-Q},\cdots ,R_{s}-\Phi^{Q-f+1}\Theta^{1-M}R_{s}^{*}\Theta^{M-1}\Phi^{-1+f-Q}]$. Likewise, the new FB SDMS-y in (12) has the following property.

**Theorem** **2.** *Assume that there are 2*K *narrowband coherent signals impinging on the URA (M×N sensors). As described in Figure 2b, the number of sensors in each row subarray is* Q*, and the new FB SDMS-y is defined in (12). If Q≥2K, then the rank of $D_{y}$ is equal to the number of the signals, namely, $rank(D_{y})=2K$.*

Based on the Theorem 2, we can divide the matrix $D_{y}$ as

(16) $$\begin{array}{c} D_{y}=\left[\begin{array}{c} D_{y1}\\ D_{y2} \end{array}\right]\begin{array}{c} \left.\right\}2K\\ \left.\right\}Q-2K \end{array}, \end{array}$$

where $D_{yQ2}=\Gamma_{y}^{H}D_{yQ1}$ and $\Gamma_{y}$ is the linear operator. By letting $a\left(v_{k}\right)={\left[1,\;e^{-j\pi v_{k}},\cdots ,\;e^{-j\pi \left(Q-1\right)v_{k}}\right]}^{T}$, the parameter $v_{k}$ can be estimated by minimizing the following cost function

(17) $$\begin{array}{c} f\left(v_{k}\right)=a^{H}\left(v_{k}\right)\Pi_{y}a\left(v_{k}\right), \end{array}$$

where $\Pi_{y}=\Omega_{y}{\left(\Omega_{y}^{H}\Omega_{y}\right)}^{-1}\Omega_{y}^{H}$, $\Omega_{y}=[\Gamma_{y}^{H},-I_{Q-2K}{]}^{H}$, $\Gamma_{y}=A_{yQ1}^{-H}A_{yQ2}^{H}={\left(D_{y1}D_{y1}^{H}\right)}^{-1}D_{y1}D_{y2}^{H}$.

### 3.3. Pair-Matching of Parameters $u_{k}$ and $v_{k}$

Since the estimated parameters $u_{k}$ and $v_{k}$ are calculated independently, the pair-matching is very important for multiple targets. In the case of *M*<*N*, by extracting the diagonal elements of URA, we can write

(18) $$\begin{array}{c} r_{diag}\left(t\right)=A\left(v_{k}\right)⊕A\left(u_{k}\right)\cdot s\left(t\right)+N_{0}, \end{array}$$

where $A\left(v_{k}\right)=A_{x}$, $A\left(u_{k}\right)$ is the submatrix of the array response matrix $A_{y}$ consisting of the first *M* rows, and $N_{0}$ is the subarray of N. Since both $A\left(v_{k}\right)$ and $A\left(u_{k}\right)$ are Vandermode matrices, $A\left(v_{k}\right)\;⊕\;A\left(u_{k}\right)$ is also a Vandermode matrix. Using the FBSS method, we can divide $r_{diag}\left(t\right)$ into *P* overlapping forward subarrays with *Q* sensors. Then, the smoothing matrix can be given as

(19) $$\begin{array}{c} D_{diag}=\frac{1}{P}\sum_{p=1}^{P}\left[R_{diag}^{p}+J_{Q}{\left(R_{diag}^{p}\right)}^{*}J_{Q}\right], \end{array}$$

where $R_{diag}^{p}=X_{p}R_{diag}X_{p}^{H}$ and $R_{diag}=E\left[r_{diag}\left(t\right)r_{diag}^{H}\left(t\right)\right]$. Hence, we can get the estimated parameters by minimizing the following cost function

(20) $$\begin{array}{c} f\left(v_{k},u_{k}\right)=a^{H}\left(v_{k},u_{k}\right)\Pi_{diag}a\left(v_{k},u_{k}\right), \end{array}$$

where $a\left(v_{k},u_{k}\right)={\left[1,\;e^{-j\pi v_{k}}e^{-j\pi u_{k}},\cdots ,\;e^{-j\pi \left(Q-1\right)v_{k}}e^{-j\pi \left(Q-1\right)u_{k}}\right]}^{T}$, $\Pi_{diag}=\Omega_{d}{\left(\Omega_{d}^{H}\Omega_{d}\right)}^{-1}\Omega_{d}^{H}$, $\Omega_{d}=[\Gamma_{d}^{H},-I_{Q-2K}{]}^{H}$, $\Gamma_{d}=A_{dQ1}^{-H}A_{dQ2}^{H}={\left(D_{d1}D_{d1}^{H}\right)}^{-1}D_{d1}D_{d2}^{H}$, and $D_{d1}$ and $D_{d2}$ consist of the first 2*K* rows and the last *Q*-2*K* rows of $D_{diag}$, respectively. From (20), the parameters $u_{k}$ and $v_{k}$ can be matched by repeating the following minimization for i=1,⋯,2K and j=1,⋯,i−1

(21) $$\begin{array}{c} \left\{i,k_{i}\right\}=\arg\min_{k_{i}}f\left(v_{i},u_{k_{i}}\right),\;subject\;to\;k_{i}\neq k_{j}, \end{array}$$

and the constraint condition $k_{i}\neq k_{j}$ can avoid the different $u_{k}$ paired with the same $v_{k}$. Then, the azimuth angle and elevation angle can be estimated as

(22) αk=tan−1ukukvkvk,θk=sin−1vk2+uk2.

### 3.4. Implementation of the Proposed Method

From (11), (17) and (22), the azimuth and elevation angles can be estimated by using the spatial differencing method, where the computational burdensome EVD is avoided and the effect of additive noise is also suppressed. Then, with the finite array data $y{\left(t\right)}_{t=1}^{L}$, the proposed method can be implemented as follows:

1. Calculate the estimated sample covariance matrix $\hat{R}$ in (4) as
  (23) R^0=11LL∑t=1Ly(t)yH(t),
  $\;LM^{2}N^{2}$ flops.
2. Form the FB SDMS-x ${\hat{D}}_{x}$ in (8) and the FB SDMS-y ${\hat{D}}_{y}$ in (12) as
  (24) $$\begin{array}{c} {\hat{D}}_{x}=\frac{1}{2P}\sum_{p=1}^{P}\left[{\hat{D}}_{xp}+J_{Q}{\left({\hat{D}}_{xp}\right)}^{*}F_{Q}\right],{\hat{D}}_{y}=\frac{1}{2F}\sum_{f=1}^{F}\left[{\hat{D}}_{yf}+J_{Q}{\left({\hat{D}}_{yf}\right)}^{*}F_{Q}\right], \end{array}$$
  where ${\hat{D}}_{xp}$ and ${\hat{D}}_{yf}$ can be calculated by using the covariance matrix ${\hat{R}}_{0}$
  $\;2Q^{3}$ flops.
3. Estimate the orthogonal projectors ${\hat{\Pi }}_{x}$ in Section 3.1 and ${\hat{\Pi }}_{y}$ in Section 3.2.
  $\;2N\left(N+1\right)Q^{2}K^{2}+2M\left(M+1\right)Q^{2}K^{2}+2\left[3Q^{2}\left(Q-2K\right)+O{\left(2K\right)}^{3}\right]$ flops.
4. Estimate the parameters $u_{k}$ and $v_{k}$ by finding the phases of the *p* zeros of the polynomial $p_{u}\left(z\right)$ and $p_{v}\left(z\right)$ using (11) and (17), where $p_{u}\left(z\right)\overset{\Delta }{\;=}z^{Q-1}a^{H}\left(z\right){\hat{\Pi }}_{x}a\left(z\right)$ and $p_{v}\left(z\right)\overset{\Delta }{\;=}z^{Q-1}a^{H}\left(z\right){\hat{\Pi }}_{y}a\left(z\right)$, $z\overset{\Delta }{\;=}e^{-j\pi u_{k}}$ or $z\overset{\Delta }{\;=}e^{-j\pi v_{k}}$
  $\;2[{\left(Q-1\right)}^{2}+O{\left(Q-1\right)}^{3}]$ flops.
5. Perform the pair-matching of the parameters $v_{k}$ and $u_{k}$ by using (18)–(21) and estimate the azimuth and elevation angles by (22)
  $\;2Q^{2}{\left(2K\right)}^{2}+3Q^{2}\left(Q-2K\right)+O{\left(2K\right)}^{3}$ flops.

As shown above, the cost of each step is roughly indicated in terms of the number of MATLAB flops. Furthermore, in the case of Q≫2K, the computational complexity of the proposed method mainly includes the calculation of covariance matrix and SDMSs, the estimation of parameters and the pair-matching, which is about $LM^{2}N^{2}+8N^{2}Q^{2}+8M^{2}Q^{2}+12Q^{3}+10Q^{2}$.

**Remark** **1.** The proposed method can estimate the parameters independently, and the null space is calculated by using the linear propagator based on the partition of the array response matrix. The polynomial roots can be obtained by the Linsey–Fox root finding algorithm, which is less than the MATLAB function roots. However, the 2D FBSS-MUSIC method in [15,16], the 2D FBSS based DOA matrix (FBSS-DOAM) method in [23], the conventional spatial differencing (CSD) method in [18,19], and the tree structure one-dimensional (1D) based (TSOD) algorithm in [22] all involve the EVD to obtain the signal subspace or noise subspace. Furthermore, as shown in Table 1, FBSS-MUSIC and CSD both need 2D spectrum peak searching. TSOD needs (2P+8K+1) 1D EVD, while FBSS-DOAM needs two times of 1D spectrum peak searching. It is easily seen that the proposed method is computationally more efficient than other recently developed methods.

**Remark** **2.** As described in (7) and (12), the proposed method can form the row and column FB SDMSs by using the auto-correlation and cross-correlation matrices among different subarrays, where we only perform the difference for the auto-correlations, and the cross-correlations are kept completely. In addition, we also employ the FB technique to improve the estimation performance. However, the TSOD method can only use the auto-correlations of different subarrays and CSD performs the difference on the whole subarrays. Thus, SDMS can achieve performance improvement over the methods in [18,19,22].

### 3.5. Cramér–Rao Bounds (CRB)

As described in Section 2, according to [24], the CRB can be obtained as

(25) CRB=σ22LReDHΠA⊥D⊕R^sT−1,

where

(26) $$\begin{array}{c} D=\left[\frac{\partial a_{1}}{\partial \theta_{1}},\cdots ,\frac{\partial a_{K}}{\partial \theta_{K}},\frac{\partial a_{1}}{\partial \phi_{1}},\cdots ,\frac{\partial a_{K}}{\partial \phi_{K}}\right] \end{array}$$

and ${\hat{R}}_{s}=\left[\begin{array}{cc} R_{s} & R_{s}\\ R_{s} & R_{s} \end{array}\right]$, $\Pi_{A}^{⊥}=I_{MN}-A{\left(A^{H}A\right)}^{-1}A^{H}$, $a_{k}$ is the *k*th column of A, *k* = 1, ⋯, 2*K*.

## 4. Simulation Results

We now evaluate the estimation performance of SDMS by using some numerical experiments. In LGA, the heights of the URA is set as *h* = 20 m and the number of sensors is *M* = *N* = 20. The colored noise is of a second-order AR model with coefficients a=[1,−0.7,−0.6] [25] and the targets are located at $\alpha =[10^{\circ },20^{\circ },40^{\circ }]$, $\theta =[20^{\circ },35^{\circ },50^{\circ }]$. The wavelength of received signals is set as 1 m and the estimation performance is examined over 300 Monte Carlo runs.

**Figure 3:**
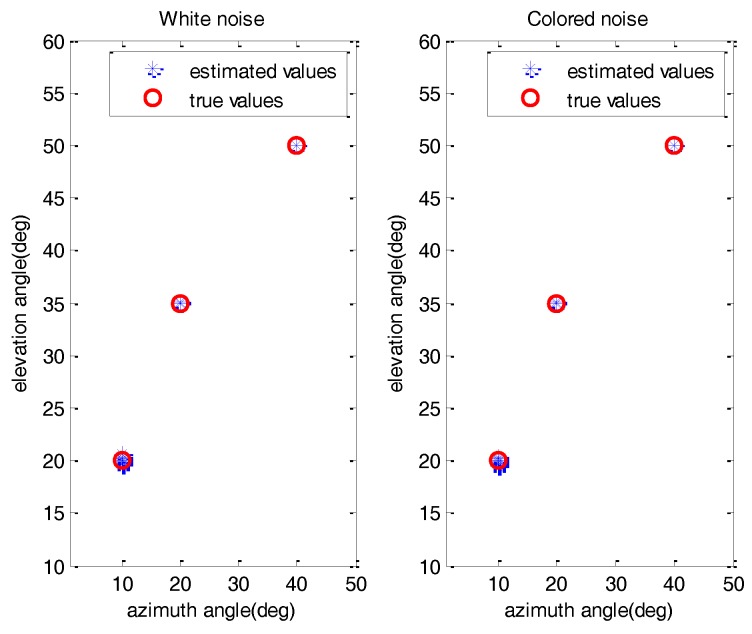
The estimated 2D DOAs of SDMS method with 100 Monte Carlo runs.

*Experiment 1*: *Effectiveness of proposed method*. In this experiment, we mainly examine the effectiveness of SDMS in the presence of white noise and colored noise conditions, where the number of sensors in each subarray is *Q* = 16 and the total number of snapshots is chosen to be *L* = 500. Here, the SNR is set as 10 dB. Figure 3 shows the estimation results of the proposed method with 100 Monte Carlo runs. As expected, all the 2D DOAs can be estimated effectively and accurately for the white noise and colored noise conditions.

**Figure 4:**
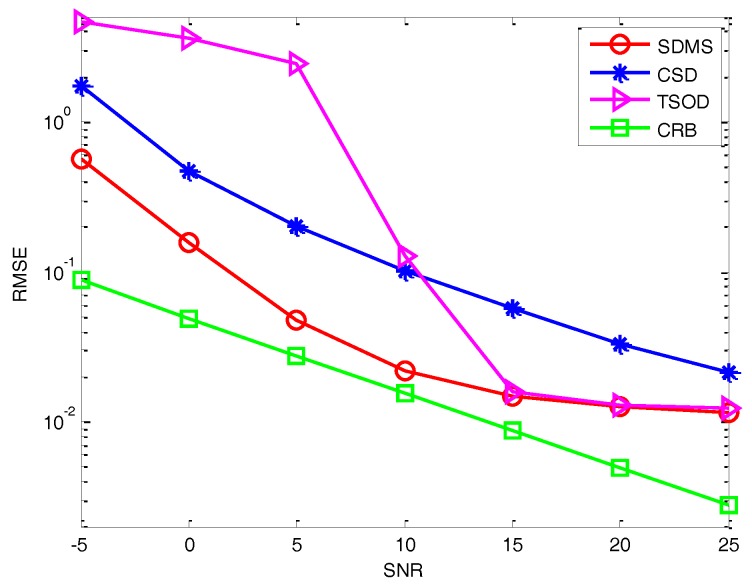
Performance versus SNR in the white noise condition.

**Figure 5:**
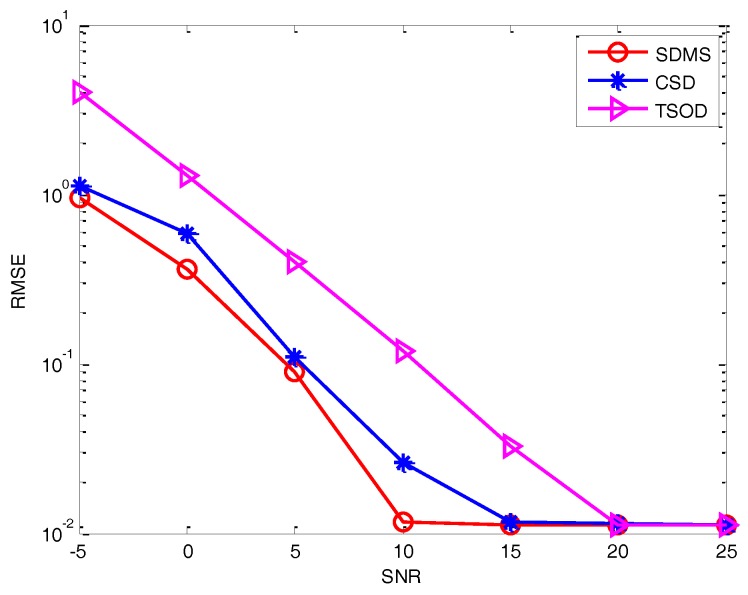
Performance versus SNR in the colored noise condition.

*Experiment 2*: *Performance versus SNR*. In Figure 4 and Figure 5, we evaluate the performance in terms of SNR in the white noise and colored noise conditions, where we assume *L* = 500 and *Q* = 16. Three methods are performed for comparison, including the CSD method in [18], the TSOD method in [22] and the proposed method. Moreover, the CRB is also provided. It can be observed that, due to the use of more information and the application of the difference operation, the performance of the proposed method is better than those of methods in [18,22]. In the white noise condition, the performance of CSD is better than TSOD for relatively low SNR, whereas it is the opposite with high SNR. It illustrates that the difference operation can reduce the effect of white noise in the low SNR condition, while the data loss will also decrease the performance in a high SNR condition. In the colored noise condition, since the non-diagonal elements of the noise covariance matrix have significant value, CSD is always superior to TSOD. Comparing SDMS and CSD, SDMS can achieve better performance by using more data information. To sum up, SDMS can achieve great performance improvement in the low SNR condition by using more information and performing the difference operation.

**Figure 6:**
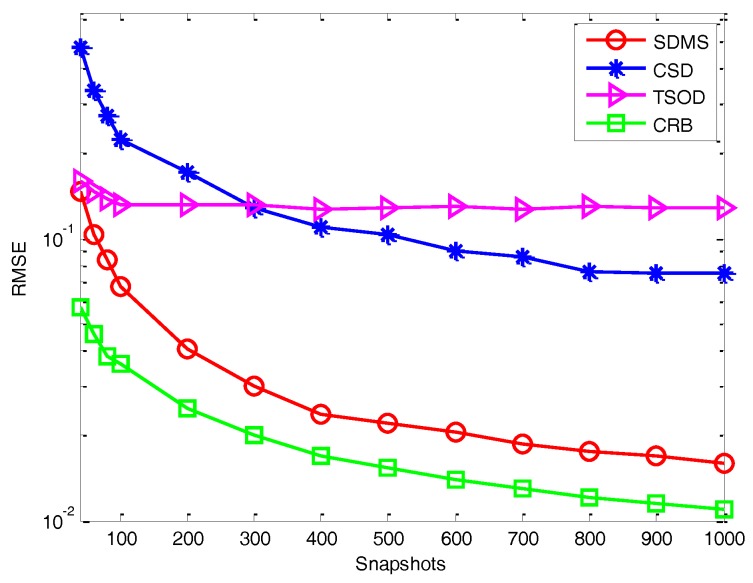
Performance versus the number of snapshots in the white noise condition.

**Figure 7:**
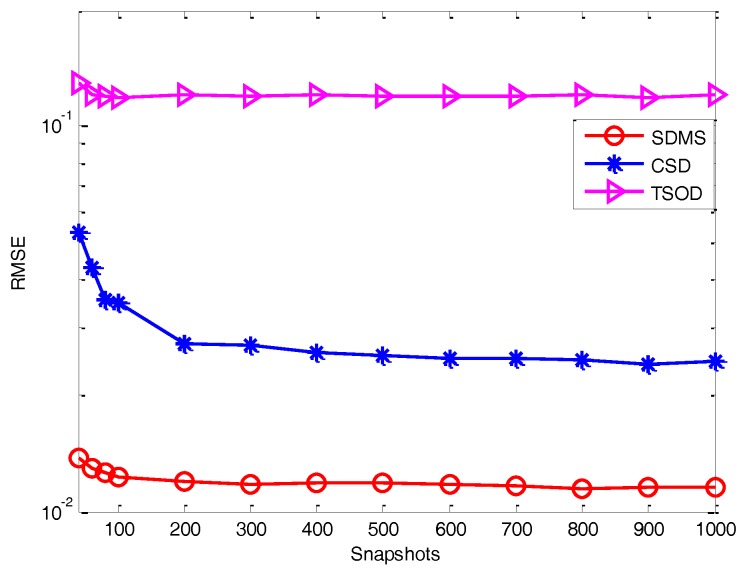
Performance versus the number of snapshots in the colored noise condition.

*Experiment 3*: *Performance versus the number of snapshots*. In this experiment, we evaluate the estimation performance with different methods (described in Experiment 2) in terms of the number of snapshots, where we assume the SNR is 2 dB and *Q* = 16. Figure 6 and Figure 7 show the RMSE versus the number of snapshots in the white noise and colored noise conditions, respectively. It is shown that, because of the use of difference operation and cross-correlation matrices, even when the number of snapshots is small, the proposed method still outperforms the methods in [18,22]. Then, for the white noise, the performance of CSD is weaker than TSOD for the small number of snapshots due to the data loss caused by the difference operation between forward and backward smoothed matrices. For colored noise, CSD performs better than TSOD. In addition, by using more data information, the performance of SDMS is also less sensitive to the number of snapshots than that of CSD.

**Figure 8:**
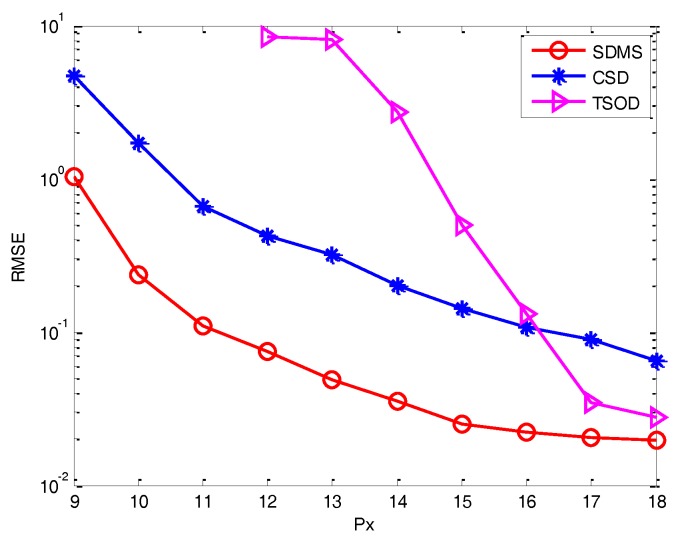
Performance versus the size of subarrays in the white noise condition.

*Experiment 4*: *Performance versus the size of subarrays*. Figure 8 describes the performance versus the size of subarrays in the white noise condition. Here, we assume *L* = 500, SNR = 10 dB. It is easily seen that SDMS still outperforms CSD and TSOD. In addition, due to the increase of array aperture, the performance of those methods becomes better. Then, we can observe that TSOD fails with a small size of subarrays but performs better than CSD with a big size. The reason is that the data loss caused by the difference operation will become larger when the size of subarrays increases. However, the proposed method can use the cross-correlation to reduce the data loss, resulting in a better performance.

## 5. Conclusions

A new computationally efficient SDMS method with little information loss is proposed to suppress the effect of white noise or colored noise in LGA. The two parameters are estimated independently by using a 1D subspace-based estimation technique, the pair-matching of which is achieved by extracting the diagonal elements of URA. Simulation results show that, in LGA, the performance of the proposed method is superior to the other recently developed method in low SNR conditions and with a small number of snapshots for white noise and colored noise conditions. In the near future, based on improving the information utilization, the extended spatial differencing method with MIMO radar for joint DOA and DOD estimation will be considered carefully.

## Figures and Tables

**Table 1:** Computational complexity comparison.

	FBSS-MUSIC	FBSS-DOAM	CSD	TSOD	Proposed Method
EVD	one, $O(Q^{6})$	two, $O(Q^{3})$	one, $O(Q^{6})$	(2P+8K+1), $O(Q^{3})$	w/o
1D searching	w/o	two	w/o	two	w/o
2D searching	one	w/o	one	w/o	w/o

## Appendix A

Based on the fact that the rank of a matrix is unchanged by a permutation of its columns [18], the rank of SDMS in (8) can be rewritten as

(A1) $$\begin{array}{c} rank\left(D_{x}\right)=rank\left\{\sum_{p=1}^{P}\left(E\left[z_{11}z_{11}^{H}\left(t\right)\right]-J_{Q}E{\left[z_{p1}z_{p1}^{H}\left(t\right)\right]}^{*}J_{Q}\right)\right\}. \end{array}$$

Then, we can calculate

(A2) $$\begin{array}{c} E\left[z_{11}z_{11}^{H}\left(t\right)\right]-J_{Q}E{\left[z_{p1}z_{p1}^{H}\left(t\right)\right]}^{*}J_{Q}=A_{xQ}\left(R_{s}-\Theta^{Q-p+1}R_{s}^{*}\Theta^{-1+p-Q}\right)A_{xQ}^{H}. \end{array}$$

Since Q≥2K, the rank of $D_{x}$ can be repressed as

(A3) $$\begin{array}{c} rank\left(D_{x}\right)=rank\left\{\sum_{p=1}^{P}\left(R_{s}-\Theta^{Q-p+1}R_{s}^{*}\Theta^{-1+p-Q}\right)\right\}. \end{array}$$

By using $R_{s}=diag\left(\rho_{1},\rho_{2},\cdots ,\rho_{K}\right)$ and $\Theta =diag\left(\Theta_{1},\Theta_{2},\cdots ,\Theta_{K}\right)$, we have

(A4) $$\begin{array}{cc} \Lambda_{p} & =R_{s}-\Theta^{Q-p+1}R_{s}^{*}\Theta^{-1+p-Q}\\ & =blkdiag\left(\rho_{1}-\Theta_{1}^{Q-p+1}\rho_{1}^{*}\Theta_{1}^{-1+p-Q},\cdots ,\rho_{K}-\Theta_{K}^{Q-p+1}\rho_{K}^{*}\Theta_{K}^{-1+p-Q}\right)\\ & =blkdiag\left(\Lambda_{p1},\Lambda_{p2},\cdots \Lambda_{pK}\right). \end{array}$$

Clearly, we can get $rank\left(D_{x}\right)=rank\left(\sum_{p=1}^{P}\Lambda_{p}\right)=\sum_{k=1}^{K}rank\left(\sum_{p=1}^{P}\Lambda_{pk}\right)$. In the matrix form, we have

(A5) $$\begin{array}{c} \sum_{p=1}^{P}\Lambda_{pk}=\Gamma_{k}V\Gamma_{k}^{H}, \end{array}$$

where $\rho_{k}=s_{k}s_{k}^{H}$, $\Gamma_{k}=\left[s_{k},\Theta_{k}^{Q}s_{k}^{*},\cdots ,s_{k},\Theta_{k}^{Q-P+1}s_{k}^{*}\right]$ and $V=diag\left(1,-1,\cdots ,1,-1\right)$. From (A5), we can prove that

(A6) $$\begin{array}{cc} rank\left(\sum_{p=1}^{P}\Lambda_{pk}\right) & =rank\left(\Gamma_{k}\right)=rank\left(\left[s_{k}^{*},\Theta_{k}s_{k}^{*}\cdots ,\Theta_{k}^{-P+1}s_{k}^{*}\right]\right)\\ & =rank\left(diag\left(s_{k}^{*}\right)\Omega_{k}\right)=2, \end{array}$$

where $\Omega_{k}=\left[\omega_{k},\omega_{k}^{*}\right]$ and $\omega_{k}=\left[1,e^{j\pi u_{k}},\cdots ,e^{j\pi \left(P-1\right)u_{k}}\right]$. Therefore, in the case of Q≥2K, from (A6), we can get that the rank of $D_{t}$ is equal to 2*K*.
